# A Simulation Study of a Novel Electrokinetic-Based Focusing Technique to Enhance the Real-Time Detection of Microplastics in Water Flow

**DOI:** 10.3390/s26113395

**Published:** 2026-05-27

**Authors:** Abdullah Abdulhameed, Yaqub Mahnashi

**Affiliations:** 1 Center for Communication Systems and Sensing, King Fahd University of Petroleum & Minerals, Dhahran 31261, Saudi Arabia; 2 Electrical Engineering Department, King Fahd University of Petroleum & Minerals, Dhahran 31261, Saudi Arabia; ymahnashi@kfupm.edu.sa; 3 Bioengineering Department, King Fahd University of Petroleum & Minerals, Dhahran 31261, Saudi Arabia; 4 Center for Biosystems and Machines, King Fahd University of Petroleum & Minerals, Dhahran 31261, Saudi Arabia

**Keywords:** microplastics, real-time detection, electrokinetic manipulation, fluid channel, 3D electrodes, non-uniform electric field, particle focusing, repulsive force, flow rate optimization, pre-concentration for sensing

## Abstract

The contamination of aquatic environments, including treated and drinking water, by microplastics poses a significant threat to ecosystems and human health. Current detection methods often rely on slow laboratory-based tests and offline analysis, which do not support real-time monitoring. This paper presents a novel focusing and concentrating device designed to enhance the real-time detection of microplastics in flowing water. The device utilizes an electrokinetic manipulation mechanism to focus microplastics toward the center of the water flow inside a pipe or fluid channel. A set of 3D rectangular electrodes, with dimensions of 5 mm × 2.5 mm × 1 mm, are arranged circumferentially and longitudinally along the inner perimeter of the fluid channel to generate an intense, non-uniform electric field. Simulation results indicate that microplastics near the channel wall experience a repulsive force on the order of $10^{-16}$ to $10^{-10}$ N toward the channel center. The applied signal amplitude and the physical properties of the microplastics strongly influence this repulsive force. The trajectories and output concentration of microplastics are investigated under varied conditions. A Voltage of approximately 25 V and a flow rate of 0.05 m/s are found to be ideal for concentrating microplastics into a narrow particle stream, enabling more efficient downstream detection and analysis. Pre-concentrating microplastics in fluid channels prior to sensing is expected to increase sensor sensitivity and improve selectivity.

## 1. Introduction

Microplastics are plastic particles in the sub-millimeter and micrometer ranges and can be found in treated water samples [1,2]. Microplastics originate from the breakdown and degradation of larger plastics, as well as industrial processes, textiles, and personal care products [3]. Microplastics’ small size and diverse chemical composition make their detection and subsequent removal through conventional methods challenging [4]. Further, while excellent at removing pathogens and large debris, traditional water treatment plants are not fully equipped to filter these tiny microplastics [5]. Even after filtration, coagulation, and sedimentation, these tiny microplastics remain in the treated water because of their size, density, and surface chemistry [6]. The conventional detection methods for microplastics in water include microscopy methods, spectroscopy methods, and flow cytometry [7]. Optical, scanning electron microscopy (SEM), and atomic force microscopy (AFM) are used to characterize microplastics by size and shape after extraction from water samples [8,9]. Spectroscopy methods, such as the Fourier-transform infrared (FTIR), shine infrared light on microplastic particles. Different microplastics absorb specific wavelengths, creating a unique fingerprint [10,11]. Raman spectroscopy is similar to FTIR, but it uses laser light to detect smaller microplastic particles accurately. However, the detection might be interfered with by fluorescent impurities. The above methods lack real-time detection, require expensive bulky equipment, and extensive sample preparation [12,13]. Other methods include microplastic fluorescent staining followed by high-throughput analysis and flow cytometry quantification [14]. Li et al. developed and validated a novel methodology that includes sample pre-treatment and flow cytometry for the analysis of microplastics without the need for staining [15]. A destructive thermal pyrolysis method and pyrolysis GC-MS (Py-GC-MS) heat the plastic until it breaks down, and the gaseous products are analyzed to identify the original polymer type [16,17]. Mathew et al. detected polyethylene microplastics (<10 μm) in wastewater effluent using curcumin as a natural fluorescent dye [18]. Portable instruments and surface-nanodroplet-decorated microfluidic devices for detecting microplastics in various water samples have recently surfaced [19,20]. However, on-site analysis still faces challenges due to environmental factors, sample complexity, and limitations in sensitivity and device miniaturization. Methods utilizing an electric field are emerging as promising alternatives to conventional techniques, offering advantages such as being real-time, lower cost, and reduced sample preparation. Colson et al. proposed an impedance spectroscopy setup for high-throughput detection of microplastics at varied concentrations and sizes [21]. They utilized the electric properties of microplastics to detect, size, and differentiate them from other biological materials at a flow rate of 103 ± 8 mL/min [21]. Zabihihesari et al. utilized DC electrophoretic force and electrical resistance measurements between two microwires in a PDMS microchannel to extract and detect microplastics in water within a 5–100 ppm concentration range [22]. They reported a positive correlation between the concentration of microplastics and the reduction in the normalized resistance recorded by the source meter. Warraich et al. presented an electromicrofluidic sensor incorporating a Wheatstone bridge and MXene-coated microwires for enhanced in situ detection of microplastics in salty water [23]. In early 2025, Fadda et al. introduced dielectrophoresis (DEP), an electrokinetic method, to enhance the detection of nanoplastics in drinking water [24]. The authors applied an electrical signal of 5 Vpp and a frequency of 1 MHz to fill the Raman confocal volume with concentrated microplastics. The above methods require the sample to be in the laboratory and lack real-time capabilities. In addition to the DEP, other methods for particle focusing are available, including hydrodynamic focusing, inertial microfluidics, and acoustic-based approaches [25,26,27]. While these techniques can be effective, they often rely strongly on flow conditions, channel geometry, or specific particle size ranges, which can limit their flexibility and selectivity. In contrast, DEP enables highly controllable, label-free manipulation with strong sensitivity to particle electrical properties, making it particularly well-suited for precise focusing and selective concentration of different types of microparticles [28]. This work presents a novel design that enables the real-time focusing and concentration of microplastics at the center of a pipe with flowing water. The device utilizes 3D rectangular electrodes arranged circumferentially and longitudinally, and connected alternately to the negative and positive terminals. The unique configuration of the electrodes enables the generation of an intense, non-uniform electric field around the inner perimeter of the channel, in the direction of the water flow. The intense electric field induced an electrophoretic force that pushed the microplastics toward the center of the flow, concentrating and focusing them before arriving at the sensing location at the outlet. The trajectories of the particles at the outlet are investigated at different signal and flow conditions. Further, the effect of the physical properties of the microplastic, such as diameter and density, and the required force to concentrate them are also investigated. The outcome of this study indicates that concentrating microplastics in real-time prior to sensing is a recommended step to enhance the sensitivity and selectivity of sensors.

## 2. Methodology

**Figure 1:**
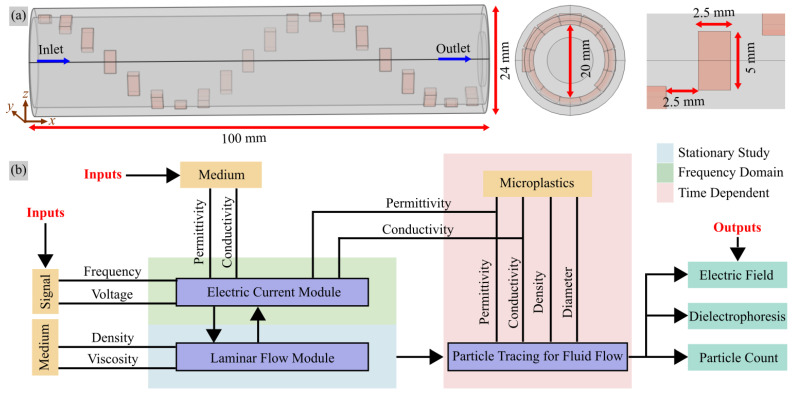
The methodology used in this work. (**a**) Structure of the simulated design. (**b**) Simulation framework.

The proposed system is a two-stage device composed of a focusing unit and a sensing unit. The focus of this paper is on designing and analyzing the focusing unit, while the sensing unit, located at the downstream end of the pipe, is outside the scope of this study. The sensing unit is based on impedance spectroscopy, employing a four-electrode array positioned at the downstream end. Two electrodes inject a small, fixed current, and the remaining electrodes measure the resulting voltage variation, from which the impedance is calculated as the ratio of voltage to current. Figure S1 in the supporting information illustrates the overall system architecture. The methodology for designing and structuring the concentration device begins by simulating and modeling the response of microplastics under varied conditions. Figure 1a shows that the device consists of a cylindrical pipe, 10 cm in length, with an inner diameter of 2 cm and an outer diameter of 2.4 cm. The electrodes have a length of 5 mm and a width of 2.5 mm, separated by a 2.5 mm spacing. Regarding the electrode thickness, thicker electrodes generate a stronger electric field toward the channel center but can obstruct the fluid flow, while thinner electrodes improve flow conditions but produce weaker electric fields at regions near the center of the channel. Therefore, a balanced electrode thickness of 1 mm was selected to ensure adequate electric field strength with minimal flow disturbance. Twenty electrodes are arranged circumferentially, longitudinally, and connected alternately to the negative and positive terminals. The novelty of this work lies in placing these electrodes in a spiral pattern on the inner wall of the tube. This pattern generates a three-dimensional spatially non-uniform electric field configuration within the center of the pipe aligned with the direction of water flow.

Figure 1b illustrates the simulation model used in COMSOL Multiphysics 6.3. The model couples three primary physics interfaces: Electric Currents to solve for the AC electric field distribution, Laminar Flow to model the fluid motion, and Particle Tracing for Fluid Flow to track the trajectories of microparticles. The electroosmotic flow, which drives fluid motion via the electrical double layer, is considered when simulating the total velocity of microplastics, including the velocity due to DEP. The electrode-fluid boundaries are set to electroosmotic velocity because the electrical conductivity of the medium is a critical factor that might result in a significant double-layer effect on the flow at low frequencies. The boundary conditions are Electric Potential conditions applied to alternating electrode pairs (±20 V peak AC voltage at 1 MHz) to create a non-uniform electric field, while Electric Insulation is used elsewhere. The selection of the above high frequency is due to the fact that at high frequencies, other electrokinetic phenomena become insignificant, such as electroosmosis resulting from the electric double layer, and electrothermal forces [29]. For the fluid flow, inlet and outlet conditions prescribe the pressure-driven flow, and no-slip conditions are applied to the channel walls. The Particle Tracing module incorporates only the DEP force, with Release conditions for particle injection and Freeze conditions at boundaries to track the trajectories of particles. The time step size is set to 5 ms, starting from 0 to 5 s. The step size is neither too large, which would lead to inaccurate trajectory calculation, nor too small, which would result in excessive computational time. Regarding the solver steps, first, the laminar flow module is solved using a stationary study. Then, a frequency-domain study is adopted to solve the electric current module, which is a frequency-dependent module. The transient study takes the output from previous studies to compute particle trajectories. The medium and microplastic properties, including permittivity, conductivity, density, and diameter, are defined in the parameters. The simulated microplastics, which differ in their chemical and physical properties, are Polyethylene (PE), Polypropylene (PP), Polystyrene (PS), Polyethylene terephthalate (PET), and Polyvinyl chloride (PVC), as listed in Table 1. These differences in the properties of microplastics allow them to have unique electrical and dielectric properties, which might be utilized to find their crossover frequency at which their motion direction can be controlled. The electrical conductivity and permittivity of de-ionized water are used for the medium. While pure water is indeed rare in natural environments, this simplification enables the study of the effects of geometry and excitation parameters without the confounding influence of salinity levels, which will be considered in future studies.

The objectives of the simulation are first to visualize the electric field distribution and the intensity of the DEP throughout the device structure. Second, it optimizes the electrode size, signal characteristic, and sample flow rate. For example, the signal amplitude is varied to determine the minimum voltage required to concentrate the microplastics in the center of the channel with a defined geometry. Third, it tracks the trajectories of the microplastics to understand the effect of their properties, including density and diameter, on their motion in the pipes.

## 3. Results and Discussion

**Figure 2:**
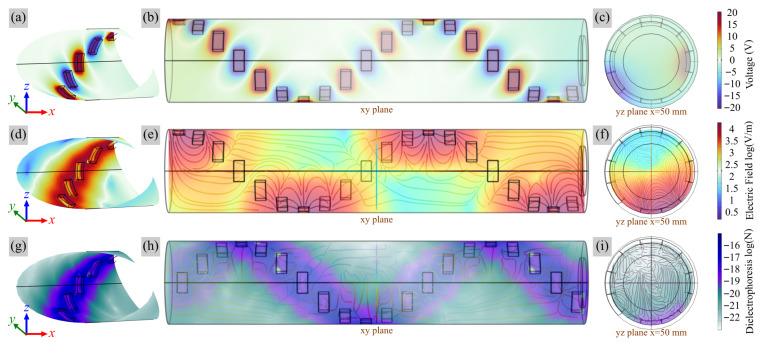
The 3D, side-view, and sectional end-view of the designed device. (**a**–**c**) Voltage potential distribution. (**d**–**f**) Electric field distribution. (**g**–**i**) DEP distribution.

Figure 2a presents the potential distribution within the pipe at an applied signal of 20 V. In the designed configuration, one electrode is held at a high potential (20 V) and another at a lower potential (−20 V) consecutively. Figure 2b illustrates how the voltage drops across the channel volume. The end-view in Figure 2c shows how the potential is distributed across the channel’s cross-section at the outlet. The electric field distribution in Figure 2d has a maximum intensity in the order of 10^4^ V/m that appears above the electrode surface and at the edges. The proposed design allows the electric field to be non-uniform, especially at the electrode gap between the electrodes and above their surfaces, where microplastics are likely to pass. The third row, Figure 2g–i, maps the magnitude of the resulting DEP force. The DEP force distribution directly mirrors the electric field gradient $\left(\nabla {\left|E\right|}^{2}\right)$ with the properties of the microplastics. The force is strongest in the regions where the spatial change in the electric field is most significant, which coincides with the electrode edges. In conclusion, the simulations confirm that the electrode geometry successfully generates the required non-uniform fields precisely where needed to focus microplastic. The concentration effect is a direct and predictable consequence of the engineered electric field.

**Figure 3:**
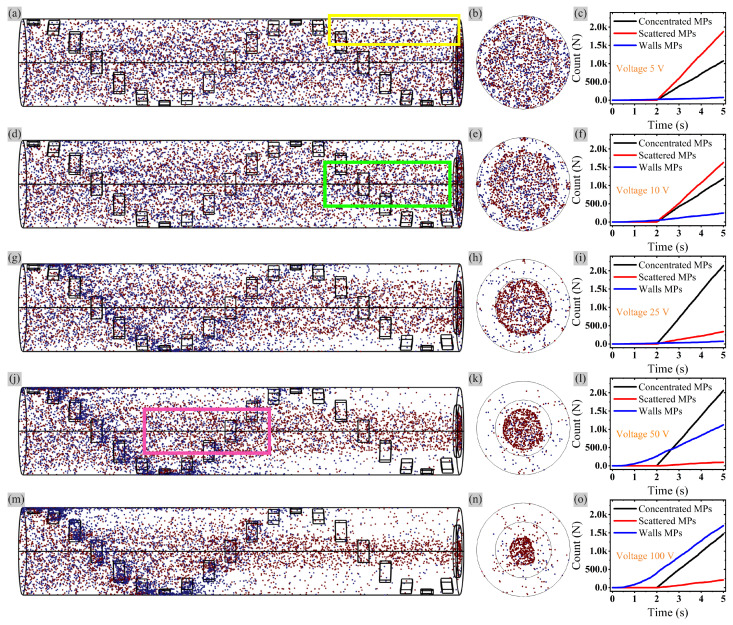
The side view, sectional view, and microplastic distribution at different signal amplitudes. (**a**–**c**) 5 V, (**d**–**f**) 10 V, (**g**–**i**) 25 V, (**j**–**l**) 50 V, and (**m**–**o**) 100 V. The red dots in the Figure are microplastics, and the blue dots represent conductive particles. Video animations are provided in the Supporting Information.

Figure 3 describes the behavior of microplastics at different signal amplitudes. It is expected that higher voltage causes intense DEP as the force is proportional to the square of the electric field. At an amplitude of 5 V, no notable change in the flow of the microplastic is observed. The MPs are widely dispersed throughout the pipe from the inlet towards the outlet, as seen in Figure 3a. The electric field is too weak to exert a significant concentration force against the flow and the random motion of MPs. There is a significant change in the distribution in the fourth quarter of the channel, where the density of the particles reduced in the region near the channel walls (yellow square). The sectional view (Figure 3b) shows a diffuse, cloudy distribution of particles, confirming they are not concentrated. Figure 3c shows that the number of scattered MPs is extremely high compared to the concentrated and wall MPs. Note that the concentrated particles are the particle count in the pipe center in a circle with a radius of 1 cm. Figure 3d shows that a slight focusing effect begins to appear as the voltage increases to 10 V. The MPs start to move away from the walls and begin to gather more in the center of the pipe. The MPs cloud in the sectional view in Figure 3e becomes slightly more defined, hinting at a central concentration (indicated by the green square). Figure 3f shows that the scattered MPs count starts to decrease, while the concentrated MPs count begins to rise, indicating a significant DEP force at 10 V. A clear, focused stream of MPs is now visible flowing through the center of the pipe and reaching the outlet when increasing the peak voltage to 25 V, as shown in Figure 3g,h. The DEP force is now strong enough to overcome the dispersive effects and gather the particles effectively. The concentrated MPs curve in Figure 3i shows a significant peak, meaning most particles are successfully focused, while the scattered MPs have dropped substantially. Unlike the previous cases, the MPs start to concentrate in the stream earlier in the first half of the pipe at a voltage of 50 V, as indicated by the pink square in Figure 3j,k. At this voltage, Figure 3l shows that the count of the concentrated MPs starts to decrease from its peak, while the wall MPs count shows a clear and sharp increase. This suggests the electric field is now so strong that it is beginning to push a significant fraction of the particles all the way to the pipe walls, where they become trapped. The MPs are excellently focused to a circle with a diameter of 5 mm at 100 V, as shown in Figure 3m,n. However, the number of concentrated MPs has fallen dramatically, while the walls MPs curve dominates, reaching its maximum, indicating excessive DEP force at 100 V. In conclusion, voltages around 25 V are ideal for concentrating MPs into a narrow stream for more efficient downstream detection or analysis. In comparison, voltages higher than 50 V are suitable for removing MPs from the flow entirely by immobilizing them on the walls for later collection or disposal. Optimizing the supplied voltage to induce DEP is critical to move the target MPs to the location of interest. Figure S2 in the Supporting Information explains how microplastic focusing enhances sensor sensitivity and selectivity. Without focusing, particles are distributed across the pipe cross-section, and only a fraction passes through the sensor’s most sensitive region, as shown in Figure S2a. However, focusing narrows the particle stream spatially and forces a much higher fraction of particles through the sensor’s optimal detection area, as shown in Figure S2b. This results in a higher signal per unit time and improved overall sensor sensitivity. The sensitivity also improved by reducing background noise. In our case, unfocused flows cause random particle-wall interactions, while focusing keeps particles away from pipe walls, reducing wall-induced noise, fouling, and parasitic signals, which may result in a cleaner baseline and thus lower detection limits. Selectivity is about distinguishing which particles matter from those that do not. Figure S2c shows that both targeted and non-targeted particles pass between impedance spectroscopy electrodes at equal relative concentration [33]. The DEP focusing targets specific particles, while particles outside the target size or physical-property range remain unfocused (Figure S2d), missing the optimal detection area or producing weaker signals. This mechanism acts as a physical pre-filter before sensing.

**Figure 4:**
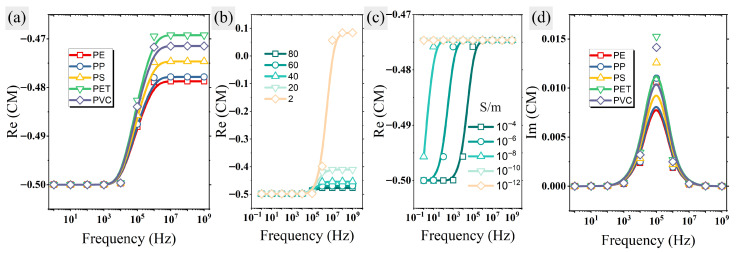
(**a**) Real part of the Clausius-Mossotti factor of different microplastics in a standard water medium. (**b**) Re(CM) of PS as a function of frequency at different medium dielectric constants. (**c**) Re(CM) of PS as a function of frequency at different medium conductivities. (**d**) imaginary part of the Clausius-Mossotti factor of different microplastics in a standard water medium.

Figure 4a shows the real part of the Clausius-Mossotti factor [Re(CM)] for the five microplastic particles in a water medium, plotted against a range of frequencies from 1 Hz to 1 GHz. The graph shows a consistently negative Re(CM) value across the entire frequency range. This indicates that the DEP force of all these microplastics in water is always negative. The microplastics will be repelled from high-field regions and pushed towards the center of the flow, which aligns perfectly with the “focusing” behavior observed in Figure 3. The higher Re(CM) of PET and then PVC is due to their higher dielectric constant compared to the other microplastics. Figure 4b shows the changes in the Re(CM) of PS as a function of frequency at different medium dielectric constants. The microplastic experiences negative DEP except at a medium dielectric constant of 2, which is less than the dielectric constant of the microplastic. Figure 4c shows that the response curve shifts to the right by one order of magnitude as the conductivity of the medium increases by one order of magnitude. This is because the conductivity affects how electric charges build up at the particle-medium interface, which is critical for real-world applications where water salinity can vary. Figure 4d is the imaginary part of the CM, which is a key for understanding the transition between positive and negative DEP, and at which frequency this transition can happen. Since the crossover frequency in this case does not indicate the transition from positive to negative, different microplastics can be tagged chemically, such as adding functional groups to their surface, or physically, such as mixing them with magnetic microparticles, to alter and modify their CM and then have further control on their DEP response.

**Figure 5:**
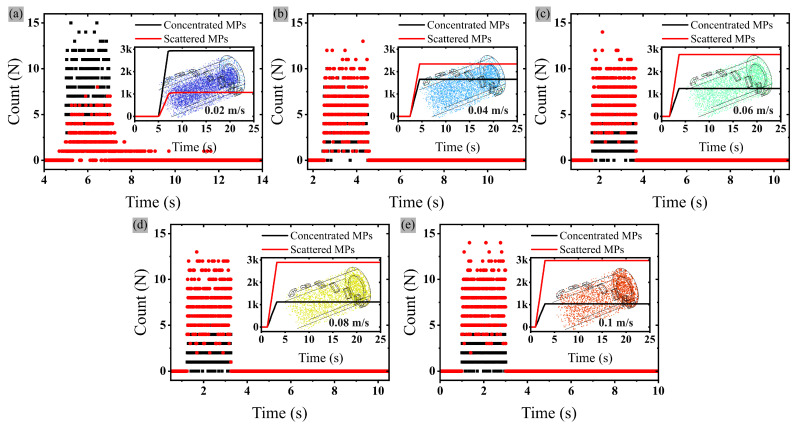
The concentration of microplastics as a function of sample velocity. (**a**) Velocity of 0.02 m/s. (**b**) Velocity of 0.04 m/s. (**c**) Velocity of 0.06 m/s. (**d**) Velocity of 0.08 m/s. (**e**) Velocity of 0.1 m/s. The inset figures show the cumulative count of microplastics over time.

The flow velocity of the sample in the pipe is an essential factor in the concentration of the microplastics. The concentration efficiency decreased with the increase in the flow velocity as the medium’s velocity overcomes the velocity of particles induced by the DEP. Figure 5 presents the concentration of microplastics under varying medium flow rates ranging from 0.02 m/s to 0.1 m/s. Figure 5a illustrates microplastics’ transient behavior and final focusing state under a medium flow rate of 0.02 m/s. The concentrated MPs, represented by black data points, clearly dominate those represented by red data points, indicating a sustained high count. The black curve in the Figure 5a inset shows that the cumulative concentrated microplastic count is higher than that of scattered particles, indicating that at slow flow rates, microplastics experience sufficient residence time within the electric field to be moved to the focusing region along the channel centerline. Note that the microplastic release time only continued for 2 s with 10 particles per 0.005 s, which explains the saturation in the cumulative curves. Figure 5b,c show that as the flow velocity increases to 0.04 and 0.06 m/s, the overall focusing efficiency begins to decrease. The scattered MPs increase while the concentrated count reduces, reflecting the fact that higher flow velocities shorten particle residence time and reduce the opportunity for MPs to be fully dielectrophorically pushed toward the focusing region. In Figure 5d,e, the difference between the concentrated and scattered microplastics is almost similar, meaning that for velocities above 0.06 m/s, the applied DEP is insufficient to move the microplastics to the focus region. Thus, increasing the supplied voltage or the channel length could be a solution to improve the concentration yield.

**Figure 6:**
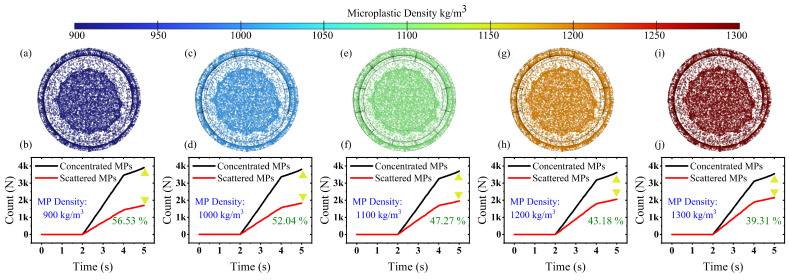
Concentration of microparticles as a function of the internal density of the microplastics. (**a**,**b**) Microplastic density of 900 kg/m^3^. (**c**,**d**) Microplastic density of 1000 kg/m^3^. (**e**,**f**) Microplastic density of 1100 kg/m^3^. (**g**,**h**) Microplastic density of 1200 kg/m^3^. (**i**,**j**) Microplastic density of 1300 kg/m^3^.

Figure 6 illustrates the distribution of microparticles as a function of their density under the application of an electric signal of 50 V and 1 MHz frequency. Although the outlet end-view shows no significant difference in the microplastic distribution, the numerical data indicate a decrease in the concentrated microplastics of 1.96–2.58% as their density increases by 100 kg/m^3^. This suggests that higher-density microplastics might require increased electric potential to trap the same number of microplastics with lower density. The figures also show the percentage difference between concentrated microplastics and those outside the focused area at the output after 5 seconds. With a density of 900 kg/m^3^, as shown in Figure 6a,b, the concentrated microplastics are more than 56% in number compared with those scattered outside the concentration region. The percentage decreases by 4% as the density increases by 100 kg/m^3^ to 1000 kg/m^3^, as shown in Figure 6c,d. The density of microplastics does not directly affect the induced DEP, as it is not a parameter in the DEP equation; the concentrated and scattered microplastics are different at different densities. This can be explained by the interplay between the gravitational and buoyancy forces. As particle density increases significantly above that of the fluid, as shown in Figure 6e–h, the downward gravitational force becomes substantial and continuously pulls particles away from the center of the flow toward the bottom wall of the channel. At a microplastic density of 1300 kg/m^3^, the percentage difference between the concentrated and scattered particles reaches a minimum of 39.31%, as shown in Figure 6i,j. For applications focusing on a specific type of MP, the flow conditions and channel geometry must be optimized to ensure that the DEP is strong enough to dominate over gravitational force for that particular density. Alternatively, operating the device in a different orientation, such as flowing the samples vertically instead of horizontally, could mitigate gravitational effects.

**Figure 7:**
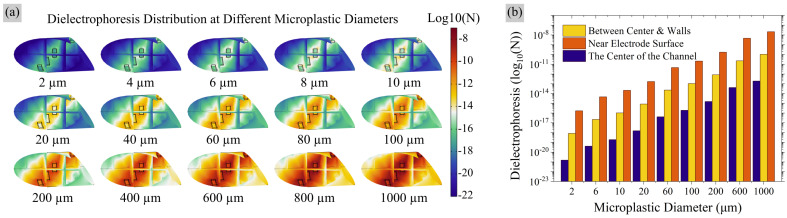
(**a**) A visual representation of the DEP force distribution across the inner pipe geometry for microplastics with a diameter range from 2 μm to 1000 μm. (**b**) A quantitative plot of DEP force magnitude as a function of microplastic diameter at different locations in the pipe.

Figure 7a visually maps the DEP force distribution within the pipe internal geometry for microplastics ranging from 2 μm to 1000 μm. The key observation is the dramatic increase in the magnitude and spatial influence of the DEP force with increasing particle diameter. The diameter range selection of microplastics is based on a previous study examining microplastic concentrations, size distributions, and polymer types in surface waters [34]. As the diameter increases, the DEP force becomes significant (r^3^ scaling) and microplastics begin to experience a measurable force that can move them away from regions of high electric field gradient, such as electrode edges and surfaces, to areas with low electric field gradient, such as the center of the electrode. Figure 7b provides a quantitative log plot of the DEP force as a function of selected microplastic diameters. The spatial variation of the electric field gradient ($\nabla {\left|E\right|}^{2}$) and its relation to the diameter of microplastics suggest two things. First, DEP is an excellent tool for size-based concentration. A carefully tuned electric field can be used to selectively concentrate or focus microplastics above a specific size threshold while allowing smaller microplastics to pass through the outlet scattered. Second, there is a fundamental challenge of using DEP for sub-micron microplastics, as the DEP force is less than $10^{-17}$ N near the electrodes and $10^{-21}$ N away from the electrode surface. Their manipulation would require extremely high electric fields or innovative electrode designs to create sufficiently high field gradients within the pipe geometry.

## 4. Conclusions

In conclusion, this study successfully designed and simulated a novel electrokinetic device for concentrating microplastics in flowing water to enable more efficient real-time detection. The unique design of the device, featuring a circumferential array of 3D electrodes, generates an intense, non-uniform electric field that repels microplastics from regions near the channel walls toward the center. Simulations confirmed that this repulsive force, which is critical to the operation of the device, is dependent on both the applied voltage and the properties of the microplastics. The investigation identified optimal operating parameters for the given device and electrode dimensions. An applied voltage of 25 V and a flow rate of 0.05 m/s were found to be effective in focusing microplastics into a narrow stream at the center of the flow. This pre-concentration step, performed before sensing, is a significant advancement, as it directly enhances the sensitivity and selectivity of downstream detection systems. Future studies include investigating the geometry of the electrodes and their distribution within the channel. Furthermore, optimizing the device parameters at higher flow rates is critical in increasing the system’s throughput. Another direction is modeling and calculating the capacitance of the microplastic at the outlet as a function of time and microplastic count. Inertial focusing with curved channels could be used to dramatically enhance the efficiency of the DEP focusing, even for smaller particles.

## Figures and Tables

**Table 1:** The properties of microplastics that are used in the simulation [30,31,32].

Microplastic	Formula	Permittivity	Conductivity (Sm^−1^)	Density (g/cm^3^)
PE	(–CH_2_–CH_2_–)_*n*_	2.2–2.4	$10^{-16}$ to $10^{-12}$	0.862–1.083
PP	(–CH_2_–CH(CH_3_)–)_*n*_	2.2–2.6	$10^{-16}$ to $10^{-12}$	0.862–1.083
PS	(–CH_2_–CH(C_6_H_5_)–)_2_	2.4–3.1	$10^{-17}$ to $10^{-14}$	1.050
PET	(–C_10_H_8_O_4_–)_*n*_	3.0–3.7	$10^{-16}$ to $10^{-13}$	1.286–1.369
PVC	(–CH_2_–CHCl–)_*n*_	2.7–3.5	$10^{-16}$ to $10^{-12}$	1.286–1.369

## Data Availability

The original contributions presented in this study are included in the article/supplementary material. Further inquiries can be directed to the corresponding author.

## Supplementary Materials

The following supporting information can be downloaded at: https://www.mdpi.com/article/10.3390/s26113395/s1, Figure S1: Structure of proposed design showing the focusing unit and sensing unit; Figure S2: Effect of particle focusing on the sensitivity and selectivity of the sensor, (a) Without focus, microplastics are scattered inside and outside the sensing area (b). After focusing, most of the targeted microplastics are concentrated in the sensing area. (c) Two types of particles are randomly distributed in the flow. (d) Focusing a specific type of particle improves selectivity. The signal that comes from the electrode represents the concentration of the focused particles only; Video S1: End view at 5 V; Video S2: End view at 10 V; Video S3: End view at 25 V; Video S4: End view at 50 V; Video S5: End view at 100 V; Video S6: Side view at 5 V; Video S7: Side view at 10 V; Video S8: Side view at 25 V; Video S9: Side view at 50 V; Video S10: Side view at 100 V.
