# Supplementary material for: A Simulation Study of a Novel Electrokinetic-Based Focusing Technique to Enhance the Real-Time Detection of Microplastics in Water Flow
Source: Sensors (Basel). 2026 May 27;26(11):3395. doi: 10.3390/s26113395 (PMC13259111; doi:10.3390/s26113395)
Supplement: Supplementary file 1 [file sensors-26-03395-s001.zip › Supplementary File.pdf]

Supplementary Data

A Simulation Study of a Novel Electrokinetic-based Focusing Technique to Enhance the Real-Time Detection of Microplastics in Water Flow

Abdullah Abdulhameed, Yaqub Mahnashi

A. Abdulhameed is with the Center for Communication Systems and Sensing, King Fahd University of Petroleum & Minerals, Dhahran 31261, Saudi Arabia (e-mail: abdullah.abdulhameed@kfupm.edu.sa).
Y. Mahnashi is with the Bioengineering Department, Electrical Engineering Department, and Center for Biosystems and Machines, King Fahd University of Petroleum & Minerals, Dhahran 31261, Saudi Arabia (e-mail: ymahnashi@kfupm.edu.sa).

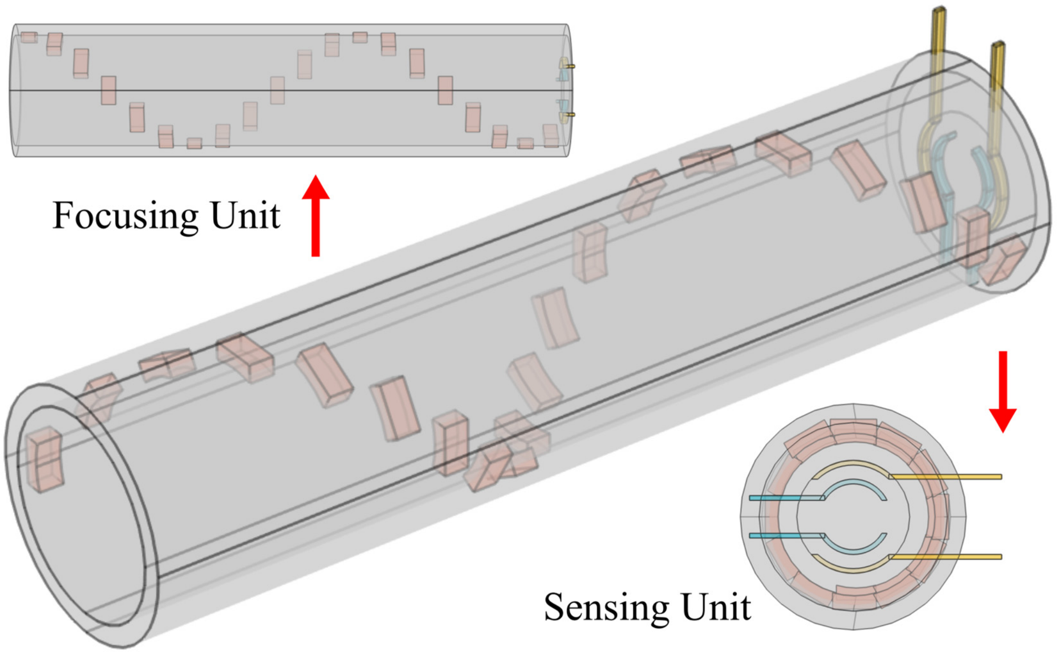

Fig. S1. Structure of proposed design showing the focusing unit and sensing unit.

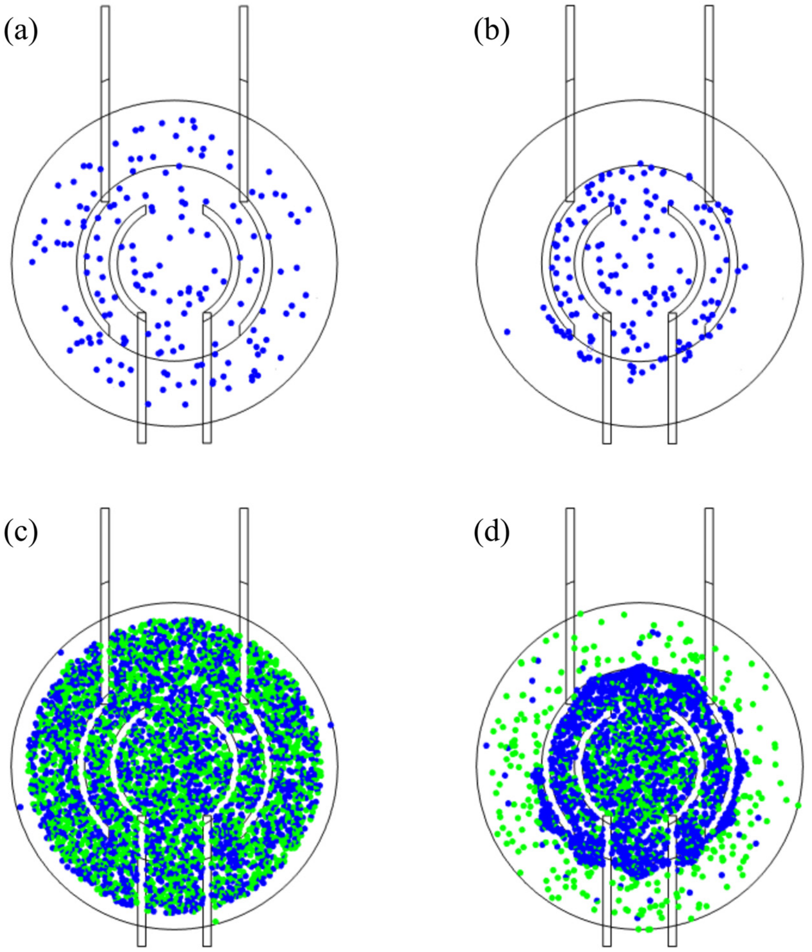

Fig. S2. Effect of particle focusing on the sensitivity and selectivity of the sensor. (a) Without focus, microplastics are scattered inside and outside the sensing area (b). After focusing, most of the targeted microplastics are concentrated in the sensing area. (c) Two types of particles are randomly distributed in the flow. (d) Focusing a specific type of particle improves selectivity. The signal that comes from the electrode represents the concentration of the focused particles only
